# Intrusion Detection System Using Deep Neural Network for In-Vehicle Network Security

**DOI:** 10.1371/journal.pone.0155781

**Published:** 2016-06-07

**Authors:** Min-Joo Kang, Je-Won Kang

**Affiliations:** The Department of Electronics Engineering, Ewha W. University, Seoul, Republic of Korea; Beihang University, CHINA

## Abstract

A novel intrusion detection system (IDS) using a deep neural network (DNN) is proposed to enhance the security of in-vehicular network. The parameters building the DNN structure are trained with probability-based feature vectors that are extracted from the in-vehicular network packets. For a given packet, the DNN provides the probability of each class discriminating normal and attack packets, and, thus the sensor can identify any malicious attack to the vehicle. As compared to the traditional artificial neural network applied to the IDS, the proposed technique adopts recent advances in deep learning studies such as initializing the parameters through the unsupervised pre-training of deep belief networks (DBN), therefore improving the detection accuracy. It is demonstrated with experimental results that the proposed technique can provide a real-time response to the attack with a significantly improved detection ratio in controller area network (CAN) bus.

## Introduction

Recently, a major advance in an automotive system has been made with integrating a number of computing devices called Electronics Control Unit (ECU). ECU is used for controlling and monitoring a subsystem of a vehicle for energy efficiency enhancement, and noise and vibration reduction. The ECU replaces conventional mechanical controlling parts [1]. More recently, automotive networking services such as Vehicle-to-Vehicle (V2V) and Vehicle-to-Infrastructure (V2I) require computing devices to perform intra-vehicular communication [2] and inter-vehicular communication [3, 4]. The vehicular communications can be applied to many practical traffic systems [5, 6]. Tang *et al*. propose to use the communications to understand driving behaviors such as each vehicle’s speed and fuel consumptions [7, 8]. Jin *et al*. show the robust V2V communications depending on a traffic stream [9]. Kesting *et al*. developed a novel message passing scheme in the communication [10]. In [11–13] efficient fuel consumptions are considered with estimating the speeds of the connected cars or their distances. Cooperative platooning enabled by the wireless communications can also improve traffic flow [14]. In the Grand Cooperative Driving Challenge (GCDC) the best performing results show the recent innovations in the fields of realistic cooperative driving [15–17]. Accordingly the ability of the computing devices in a vehicle dramatically increases.

Different communication protocols are developed to support the communication [4]. Among the protocols, Controller Area Network (CAN) [18] as the de factor standard of in-vehicle network communication is such a simple communication protocol supporting to connect sensors and actuators with ECUs, and the adoption of CAN facilitates emerging automotive applications [19]. Quite often important information such as diagnostic, informative, and controlling data is delivered through a CAN bus to serve the automotive services such as self-driving and advanced driver assistance systems (ADAS). The information must be secured for the safety of a driver. However, the growth of networking capability is accompanied with significant security concerns, and unfortunately the in-vehicular network includes several security flaws [20–23]. ECUs can obtain any ECU-to-ECU broadcasting messages in the same bus, and they are unable to identify a sender [20]. It is shown in how faked packets can confuse critical components securing driver’s safety by malicious attacks such as a packet injection and data manipulation [21–23].

There have been several research works considering safety problems in inter and intra vehicular communications [24–29]. In particular, an intrusion detection sensor (IDS) gains much attention due to the efficiency and simplicity in detecting the attacks [24–27]. Hoppe *et al*. propose an intrusion detection method by using several representative attack patterns predefined in a database [25]. Larson *et al*. develop a specification-based approach, comparing the behavior of the current specification system to the designated patterns [26]. In [27], a sensor-based detection method recognize a malicious intrusion by using several sensors designed for the attack scenarios. Secured protocols in accordance with the conventional specifications are proposed in [28, 29].

The previous intrusion detection methods may be effective only for specific threat models that have been already considered in design stages [30, 31]. To cope with the problem machine learning based IDS techniques are employed, mainly, for conventional communication networks [32]. The idea is to capture underlying statistical features of data and use them to detect any malicious attack [33]. Intrusion detection methods using artificial neural network (ANN) [34, 35] and support vector machine [36] are developed for classifying attack types. The advanced machine learning algorithms are barely used for a vehicular network because the computing power of the conventional ECU is limited to process the complex process. However, the computing power of ECU has been notably increasing to process enormous real-time tasks in the most recent vehicular system [19].

In this paper, an intrusion detection system using the deep neural network (DNN) structure [37] is proposed to secure the in-vehicular network, *e.g*. CAN network. The proposed technique trains high-dimensional CAN packet data after the dimension reduction to figure out the underlying statistical properties of normal and attack packets, and, in defense, it extracts the corresponding features to identify the attack. DNN has been shown to be effective for classifying statistical patterns and mapping complex non-linear input-to-output relations in various research fields such as artificial intelligence, multimedia processing, security [37–40] as well as in intelligent vehicular systems [41–44]. Our work is the first to employ the deep learning structure in the IDS of in-vehicular networks, which differs from earlier ANN-based intrusion detection methods [34, 35]. Specifically, we use unsupervised deep belief network (DBN) pre-training methods [45] to efficiently train the parameters initializing the deep neural network. The parameters are tuned later to achieve a better classification result with the supervised learning. Experimental results demonstrate that the proposed method yields a superior performance in terms of a classification error with little computation complexity in the decision.

## Related Work

### CAN

CAN is designed for half-duplex and high-speed broadcast bus in-vehicular network, providing the communication rate up to 1Mbps [18]. The CAN protocol is widely used in automotive manufactures as the de factor standard. In the protocol, each ECU broadcasts a message to the network using a data packet. A sender ECU may include its unique ID number in the packet, and a receiver ECU retrieves the packet by identifying the ID of the sender. Thus CAN packet has no explicit destination field.

**Fig 1:**
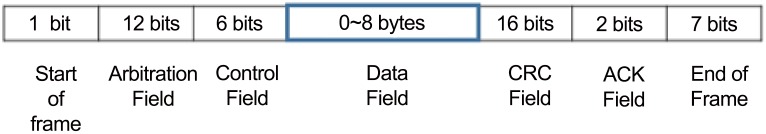
CAN packet syntax.

Fig 1 shows the syntax of the CAN data packet. The arbitration field includes an 11 bit ID field where each ID corresponds to a specific ECU. The arbitration field offers two functions: (1) prioritizing a message by the ID in the decreasing order and (2) enabling each ECU to filter an interesting message. The ID field is used for a collision avoidance algorithm in the bus, which is extended to 29 bits later. The data field contains maximum 8 bytes information to be transmitted in a message, for example, the value of the steering wheel angle and the on/off status of components in display panel. The control field contains the size of the data field. The cyclic redundancy check (CRC) field detects any error in the data packet. The acknowledgement field confirms the receipt of a valid CAN packet.

### Intrusion Detection with Machine Learning

Intrusion detection techniques have been actively studied to help the conventional network resist malicious attacks. In literature quite a number of the intrusion detection techniques are developed based on machine learning techniques, based on the assumption that the patterns of the attack packets differ from those of the normal packets. In [34–36] artificial neural networks (ANN) and support vector machine (SVM) are applied to the intrusion detection, using a statistical modeling on a packet data. In [46] a frequency-based encoding method is used for a packet feature in ANN and SVM. The aforementioned works are based on supervised machine learning techniques, and, thus a number of labeled data sets are required in the training. As compared to the approach, Kayacik *et al*. employ an unsupervised machine learning technique such as a self-organized feature map (SOM) for network intrusion detection.

**Fig 2:**
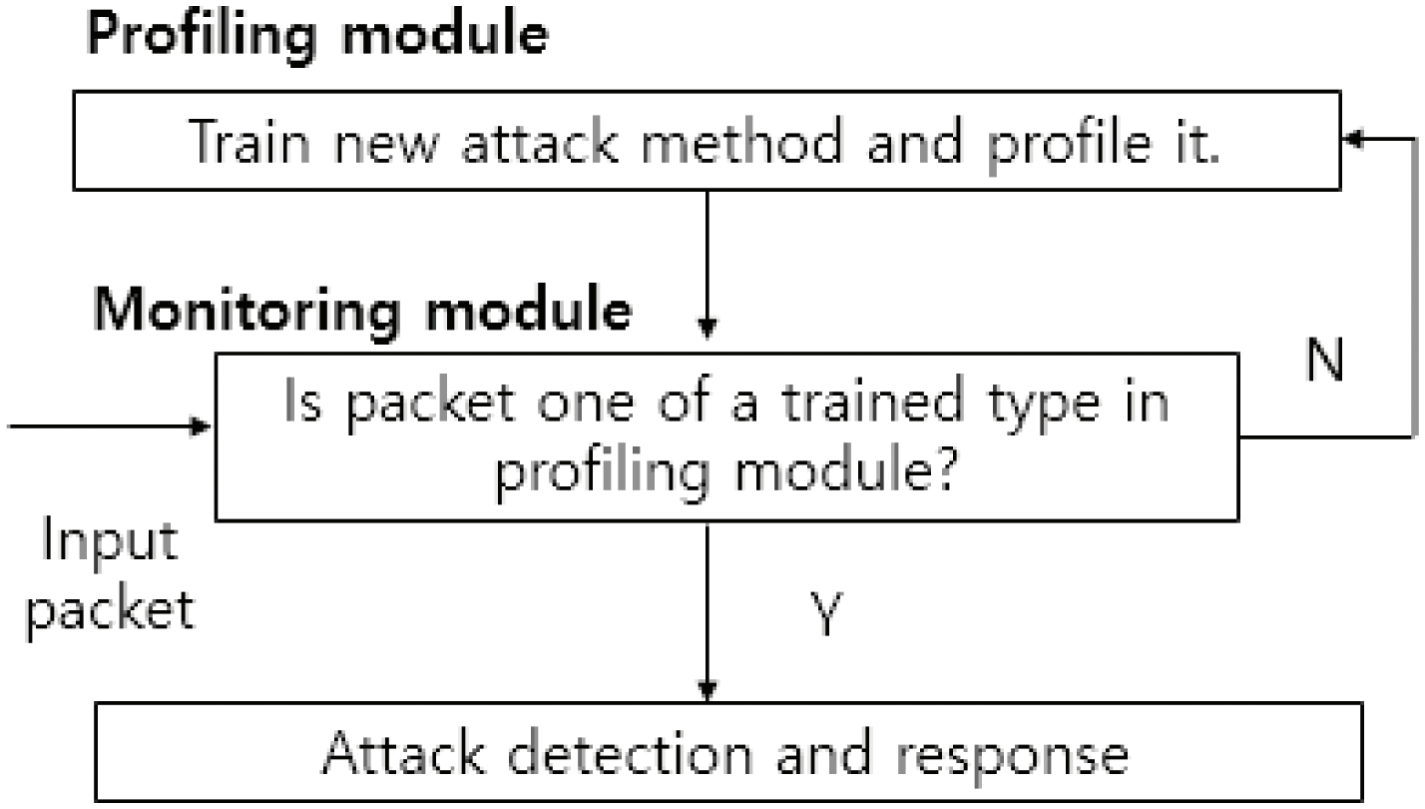
Architecture of IDS based on machine learning techniques.

Fig 2 shows a common architecture of the IDS based on machine learning. The IDS includes various modules for gathering and analyzing a large amount of data packets. Typically, the monitoring module detects a type of an incoming packet after feature extraction. The profiling module contains the features trained off-line. If the monitoring module identifies a new attack type, the profiling module may update the database of the profiling module for upcoming packets.

### Deep Learning for Classification

Deep learning refers to a machine learning technique using an architecture comprising a number of hierarchical layers of non-linear processing stages. The architecture can be categorized into two types, *i.e*., a discriminative deep architecture and a generative deep architecture, depending on how the architectures are exploited [47]. The discriminative deep architecture provides abilities for pattern classification with the supervised learning as in the conventional feed-forward artificial neural networks (ANN). The deep structure, namely, deep neural network (DNN) can be augmented with multiple hidden layers from the ANN structure.

**Fig 3:**
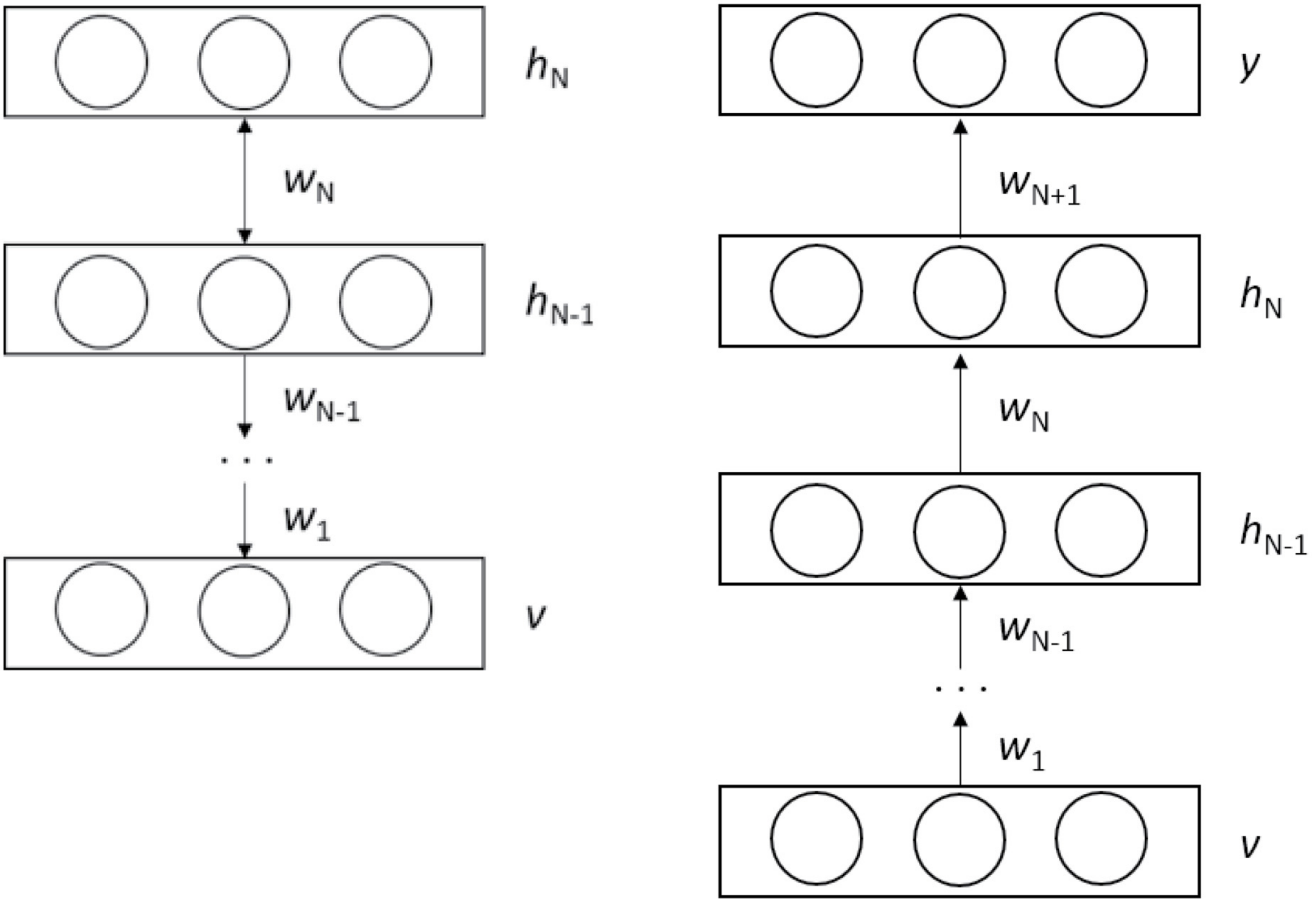
(a) DBN structure with *n* hidden layers built with a top-down manner and (b) DNN structure involving the pre-trained wight parameters in *n* hidden layers built with a bottom-up manner.

However, the augmented neural networks are inefficiently trained using the back-propagation learning with a gradient descent optimization due to the vanishing gradient problem [48]. In the backpropagation, the gradient of the error surface is computed in each layer while the gradient exponentially decreases with the number of the layers, thus causing a extremely slow convergent speed. To prevent the problem, the generative deep architecture characterizing the correlation of the observed data and the associated classes is used for initializing parameters of the discriminative architecture [49], called the unsupervised pre-training scheme. In [49], the weight parameters interconnecting nodes in adjacent layers are efficiently trained using a top-down approach by considering the nodes as restricted Boltzmann Machines (RBM). After the pre-training, fine-tuning is performed using the gradient descent method with the supervised learning as in the conventional feed-forward ANN [50]. The deep belief networks (DBN) [45] as a probabilistic generative model include several layers of stochastic hidden units on top of a single bottom layer of observed data to efficiently solve the vanishing gradient problem [49, 50]. The DBN structure is shown in Fig 3(a) where the top-two layers contain undirected connections, and the lower layers contain directed connections to the layers below. In this top-down manner, the weight vector *w*_*n*_ is generated to form the visible data vector *v*, and the set of *w*_*n*_ is used for initializing the parameters of the proposed classifiers later. The solution is used similarly for many practical applications [41, 43, 51] using the DBN learning structures, and, therefore adopted in the proposed technique to pretrain the parameter as well.

## Proposed Technique

### Proposed Intrusion Detection System with Deep Neural Network Structure

**Fig 4:**
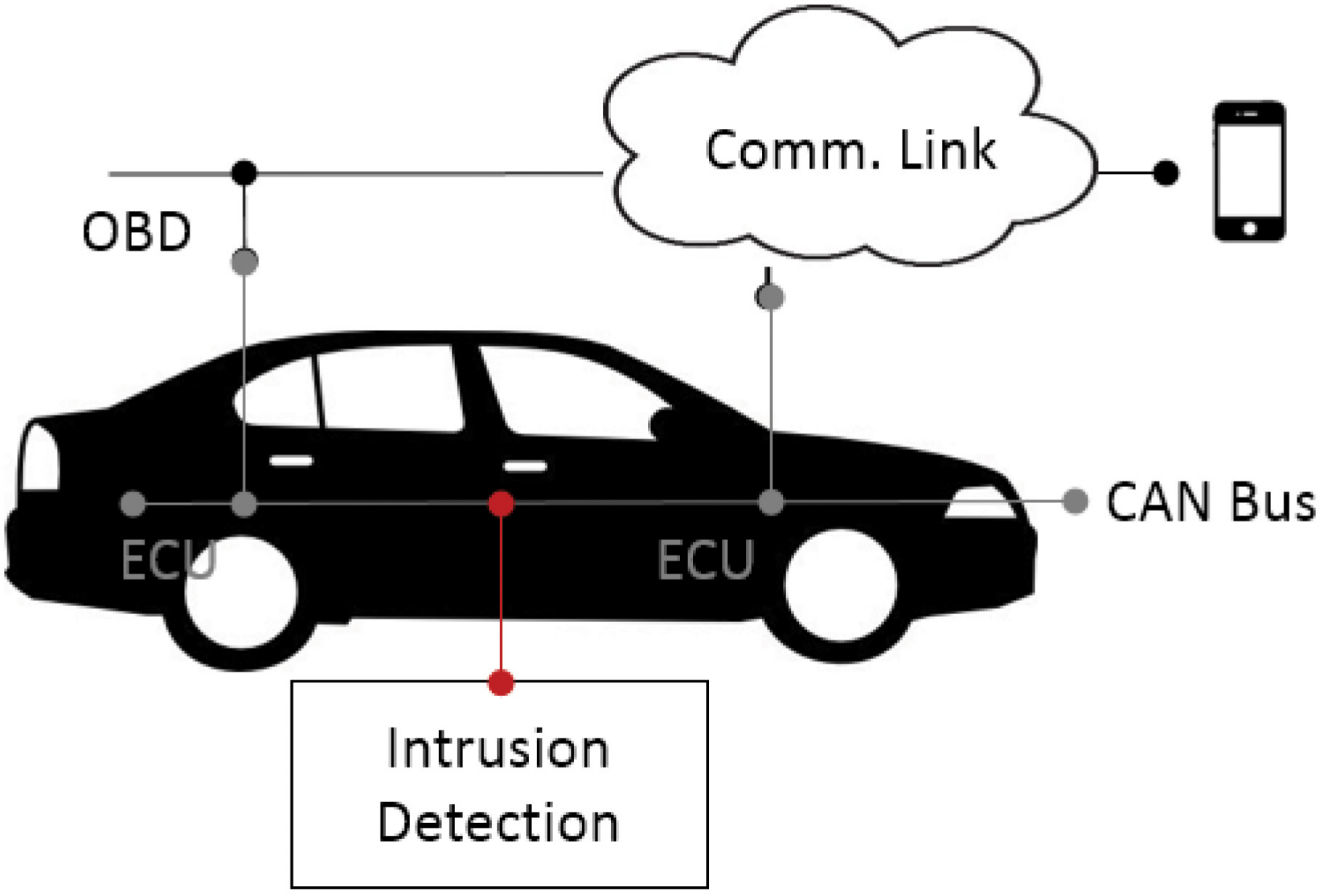
Attack scenario in the connected car.

The proposed intrusion detection system considers a general type of an attack scenario where malicious data packets are injected into an in-vehicle CAN bus. In-vehicular networks are accessed from the mobile communication links [20] such as 3G, 4G, and WIFI or a self-diagnostic tool such as OBD paired with the driver’s mobile device [29]. The proposed intrusion detection system monitors broadcasting CAN packets in the bus and determines an attack, as shown in Fig 4.

**Fig 5:**
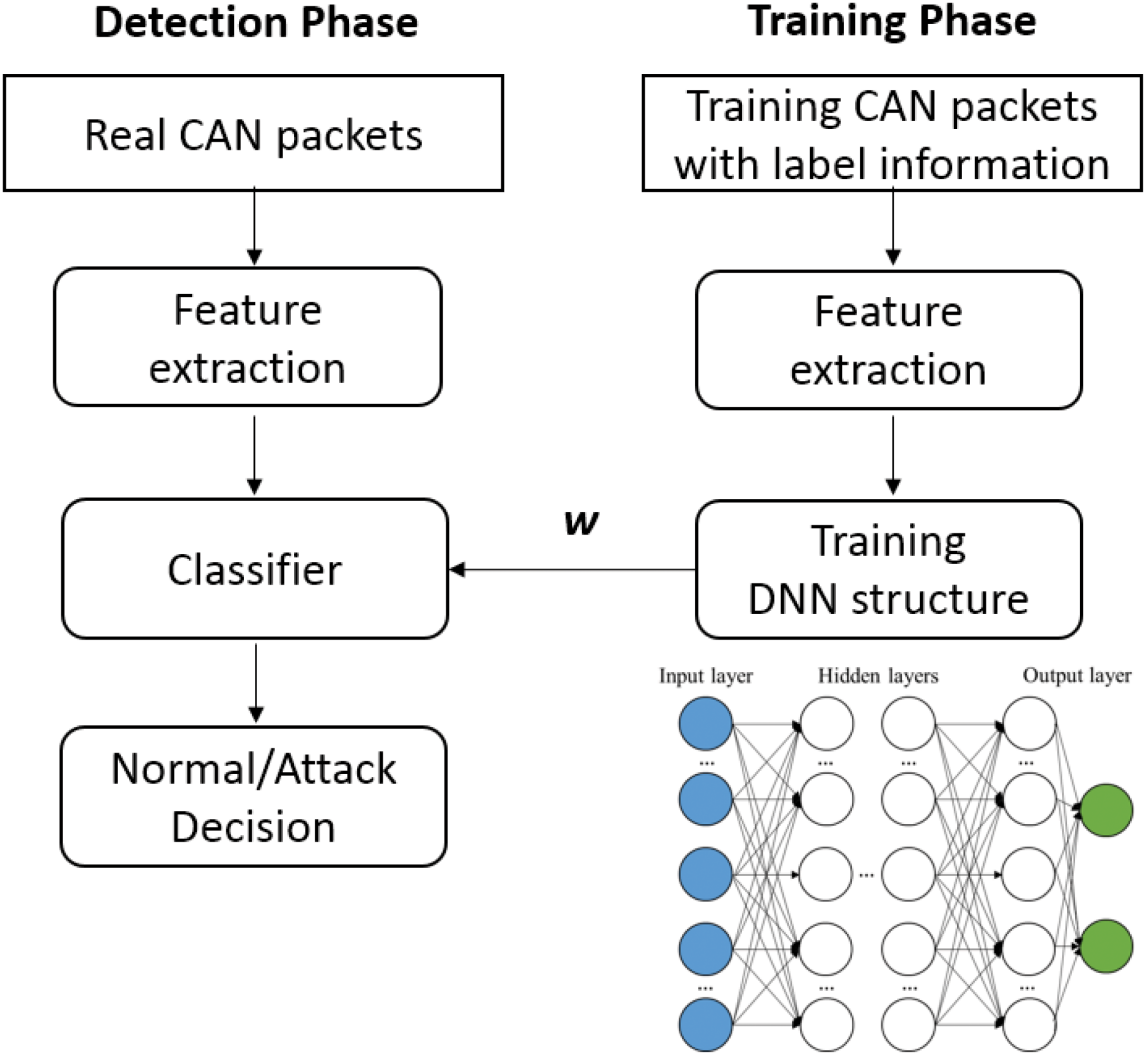
Overview of the proposed intrusion detection system.

Our IDS design consists of two main phases, *i.e*., the training phase and the detection phase as in the conventional machine-learning based IDS, as shown in Fig 5. The training phase is performed off-line as the training is time-consuming. In the training phase a CAN packet is processed to extract a feature that represents a statistical behavior of the network. Each training CAN packet has its binary label, *i.e*., either a normal packet or an attack packet in supervised learning. Thus the corresponding features are expected to represent the label information. We adopt the DNN structure to train the features, in which the weight parameters on the edges connecting the nodes are obtained. The detection phase is also shown in Fig 5. The same feature is extracted from an incoming packet through a CAN bus, and the DNN structure computes with the trained parameters to make the binary decision.

The learning structure should be configured for the supervised learning as the DBN model in Fig 3(a) provides unsupervised learning mechanism. To this aim, the final classification layer including label information is added to the top layer of the DBN model to construct the discriminative deep learning structure. Fig 3(b) shows the modified structure into the deep feed-forward ANN structure where the structure is trained with the bottom-up supervised learning manner, owing to the label information *y*. It is highlighted that the weights *w*_*i*_ in the hidden nodes of the DBN structure are obtained from the unsupervised pre-training at first. However, the parameters are used only for initializing the weights, and, they are fine-tuned by using the gradient descent method in the deep feed-forward ANN structure later.

### CAN Packet Feature

CAN feature is an abstract representation of a CAN packet. The feature is designed by considering computational efficiency. In other words, the feature is extracted directly from a bitstream of a CAN packet so that the decoding is not necessary during the extraction. The occurrences of bit-symbols in a data packet are taken into an account. In particular We choose the *DATA* field that includes 64 bit positions (= 8 Bytes) in the CAN syntax and investigate the probability distributions of the bit-symbols. Mathematically the data vector $p_{o}\in R^{64}$ is given as,

$$\begin{array}{c} p_{o}=\left\{P\left(b_{0}\right),P\left(b_{0}\right),\ldots ,P\left(b_{63}\right)\right\}, \end{array}$$ (1)

where *P*(*b*_*i*_) is the probability of a bit-symbol “1” observed in the *i*-th bit position, and

$$\begin{array}{c} p=L(p_{o}), \end{array}$$ (2)

where the function $L:R^{64}\to R^{64}$ is the logistic function: if *P*(*b*_*i*_) is greater than a half, the probability is mapped to 1. Otherwise, it is mapped to 0.

**Fig 6:**
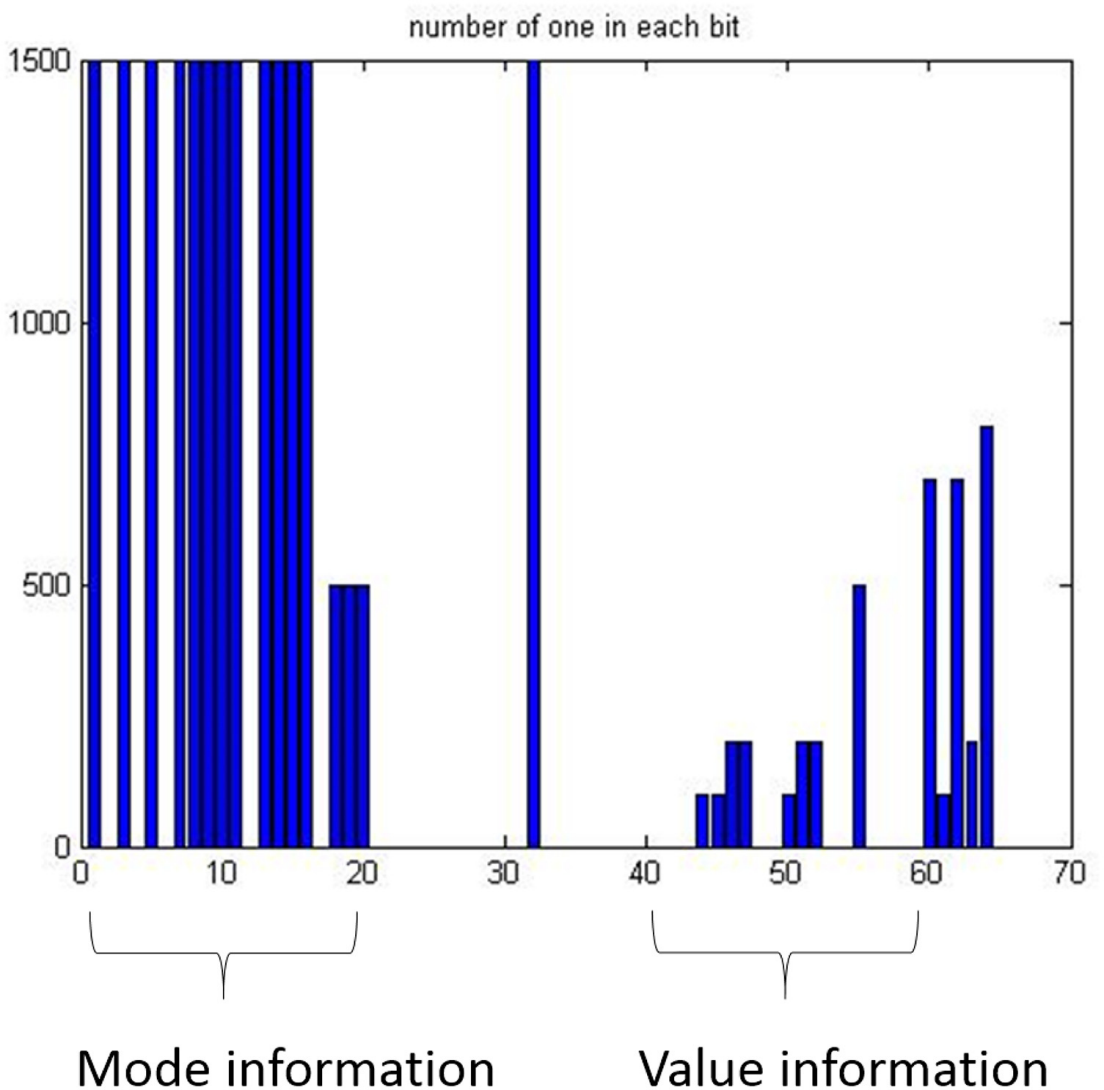
The occurrences of a bit-symbol “1” in the *DATA* field of 8 Bytes, consisting of mode information and value information, at time *t*.

All the bit positions in the *DATA* field may be used for generating the feature. However, the dimension can be reduced by considering specific semantics in the corresponding syntax element. The proposed technique regards mode information and value information according to the semantics. The mode information represents a command state of an ECU, for example, controlling wheels, and the value information represents the value of the mode, for example, the wheel angle or the speed, as shown in Fig 6. The mode information is constant in a short period, while the value information may change with some noises. In the proposed technique the value information is only used for the training phase. The usage of the mode information will be shown in the detection phase.

Denote **p**_*v*_ is the data vector reduced from **p**. Then the feature vector **v** at time instance *n* is generated as,

$$\begin{array}{c} v\left(n\right)=p_{v}\left(n\right)⊕p_{v}\left(n-1\right), \end{array}$$ (3)

where ⊕ is an exclusive-or operator applied to each position of bits in the vector.

### Training the Deep Neural Network Structure

**Fig 7:**
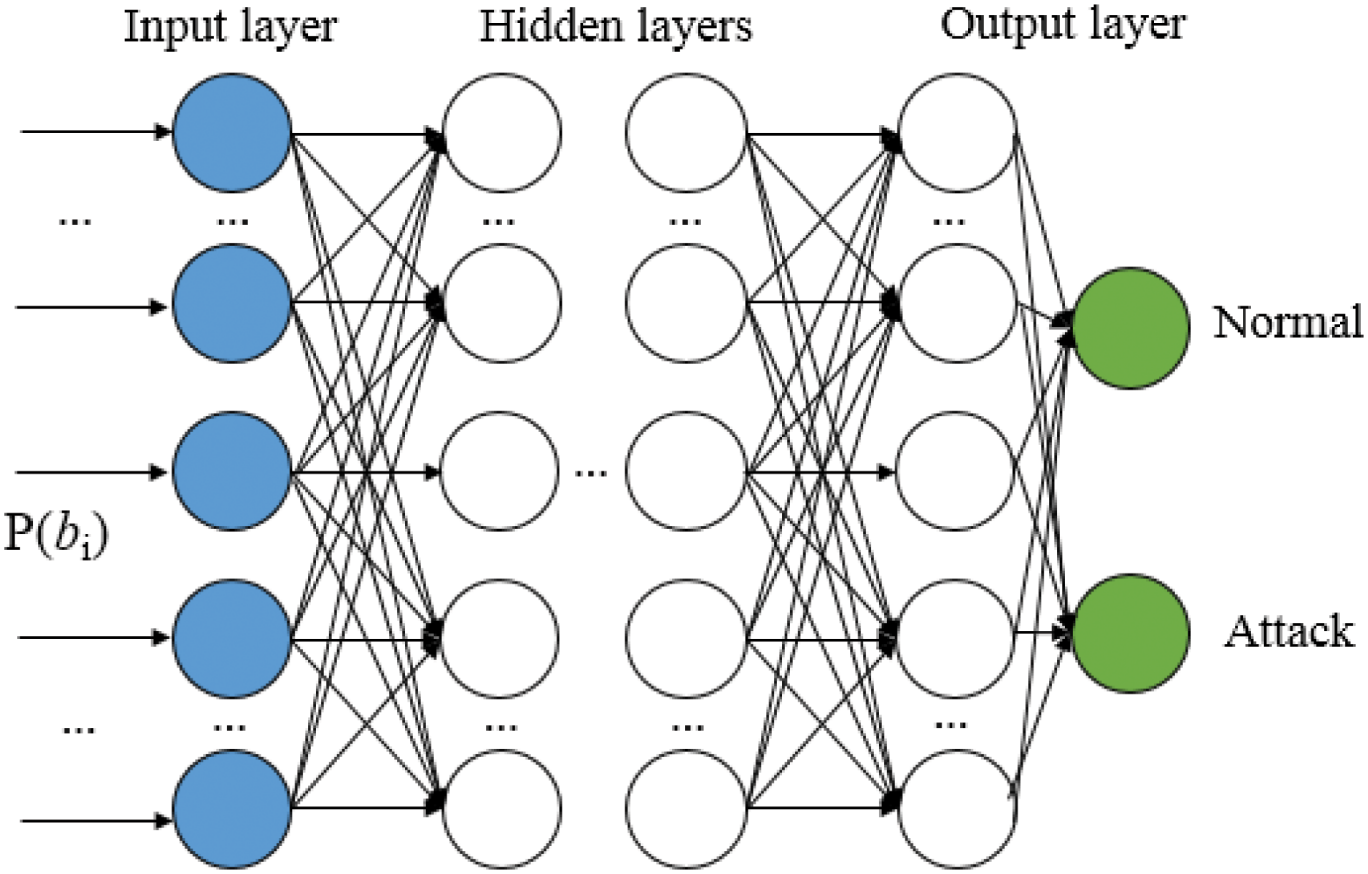
Deep neural network structure in the proposed technique.

The learning mechanism of the proposed DNN structure to classify a normal packet and an attack packet is explained. Fig 7 shows an input layer, multiple hidden layers, and an output layer. The feature vector is inputted to the input nodes of the structure. Each node in Fig 7 computes an output with an activation function using rectified linear unit (ReLU), and the linear combinations of the outputs are linked to the next hidden layers.

Considering a supervised learning, there is a training set {(**v**^1^, *y*^1^), (**v**^2^, *y*^2^),…, (**v**^*K*^, *y*^*K*^)} of *K* samples. The data vector **v** is the feature vector consisting of the probability of the bit-symbol “1”, and *y* is the binary label information, assigned to each training sample. In the learning phase, the input feature **v** goes through the visible nodes at the bottom of the neural network structure, in which initial weights are given by the DBN learning. Then, the weight vectors are fine-tuned in sequel. For this, we minimize a cost function *C* given as the mean squared error function between the prediction value and the output:

$$\begin{array}{c} C\left(w;v,y\right)=\frac{1}{2}∥h_{w}\left(v\right)-y{∥}^{2}, \end{array}$$ (4)

where **w** is the set of the weights in the network to be trained, *y* is the label, and *h*_*w*_(**v**) is a hypothesis function yielding an estimated output. The overall cost function for a batch training is defined as

$$\begin{array}{c} C\left(w\right)=\frac{1}{K}\sum_{k}C\left(w;v^{k},y^{k}\right)+\frac{\lambda }{2}\sum_{n}^{N}\sum_{i}^{M_{l}}\sum_{j}^{M_{l+1}}{\left(w_{ji}^{n}\right)}^{2}, \end{array}$$ (5)

where *N* is the depth of the neural network, *M*_*l*_ is the number of the nodes in the *l*-th layer, and $w_{ji}^{n}\in w$ is the weight of the edges between a node *i* in the layer *n* − 1 and a node *j* in the layer *n*. We want to obtain the optimal parameter set w* to achieve the minimization of the objective function as follows:

$$\begin{array}{c} w^{*}=\arg\min_{w}C\left(w\right), \end{array}$$ (6)

which can be achieved by the back propagation algorithm. In the back propagation algorithm the weight vectors are updated from the top layer to the bottom layer by using the stochastic gradient method,

$$\begin{array}{c} w_{ji}^{n}=w_{ji}^{n-1}+\zeta \frac{\partial }{\partial w_{ji}^{n-1}}C\left(w\right), \end{array}$$ (7)

where *ζ* is an adaptation parameter.

### Attack Detection

The class of a testing CAN packet is predicted in the detection phase. The output is computed with the trained weight parameters and the feature set extracted from the testing CAN packet as in the training. The classifier provides the logistic value 0 or 1, telling if the sample is normal packet or the attack packet, respectively.

**Fig 8:**
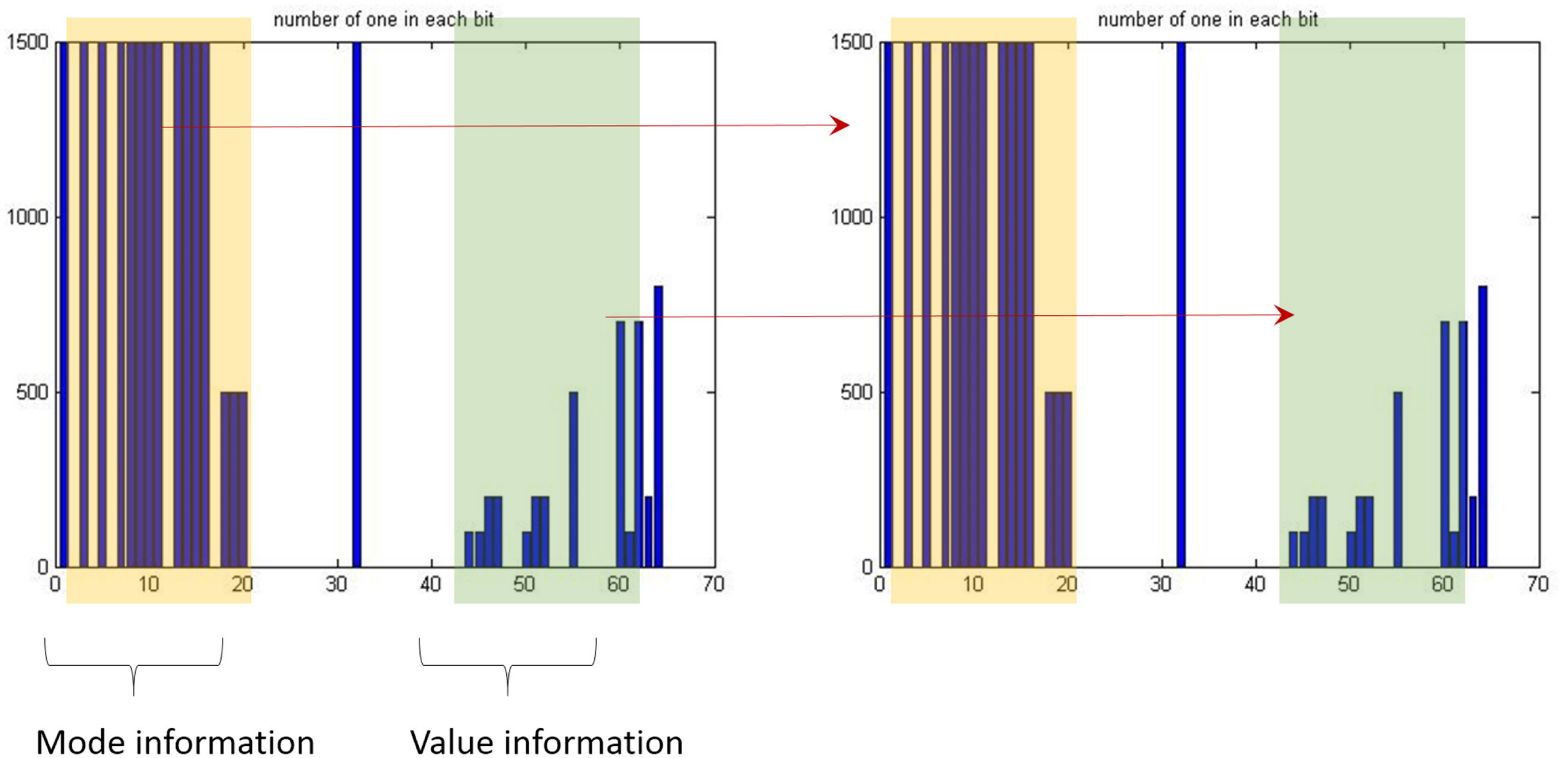
Template matching method to find the proper trained parameters.

There can be a number of attack scenarios considered in an ECU, and the weight vectors can be trained fitted to each scenario. The mode information is used for identifying the scenario in the proposed method, so that the appropriate training set can be applied. For this, template matching is developed in the proposed method. The template comprising the mode information refers to the information along with the training samples used for the specific scenario. Fig 8 shows an example of the template matching where the template is colored with yellow. As shown, if the template is matched between in the training sample and in the CAN packet to be tested, the detector uses the corresponding trained parameters obtained from the value information.

## Experimental Results

### Data Set

**Fig 9:**
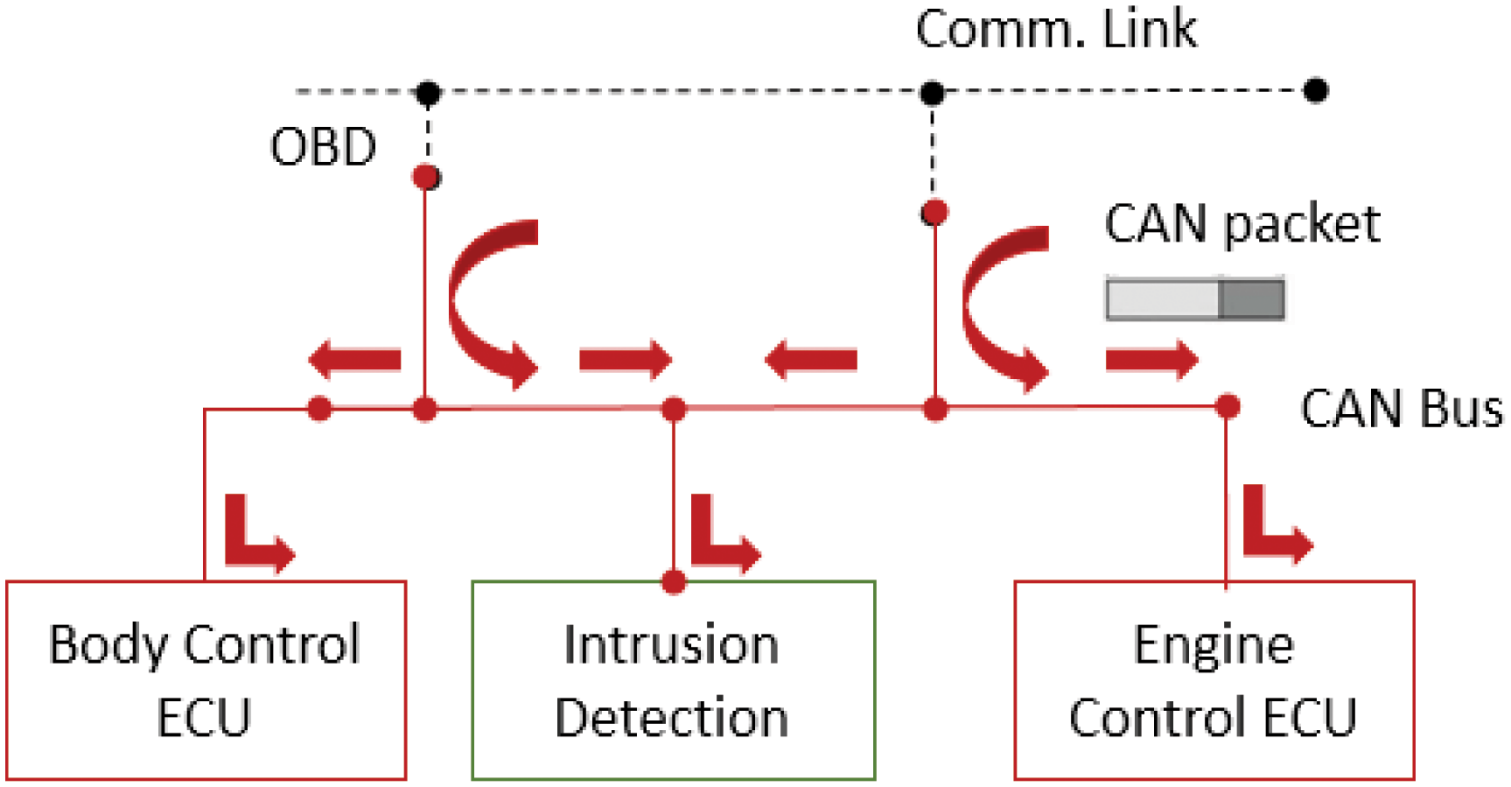
Simulation configuration.

We simulate the in-vehicular network communicating with several ECUs and the packets in the CAN bus as shown in Fig 9. The packets are created by the packet generator named Open Car Test-bed and Network Experiments (OCTANE) [52] in the simulation, and they are sent to the CAN bus. Our IDS monitors the network packets. The number of the generated packets is about 200,000 in a simulation. To avoid the over-fitting problem, we assign 70% packets to the training data and 30% packets to the testing data. In the attack scenario some of the packets are injected and are manipulated to deceive the system. Note the attack packets are inserted with some time intervals, so that they are not burst in the in-vehicle network.

Table 1 shows the CAN data packets including the identifiers (ID) and *DATA* fields to control ECUs, considered in the experiment. Because each ECU has a unique ID, the data packet can be generated for the corresponding ECU. The constant bit fields in the packet syntax are used for the mode information while the variables denoted by *α*_*i*_ *β*_*i*_ are used for the time-varying value information. Furthermore we add a Gaussian noise into the value information to give randomness.

**Table 1:** CAN packets used in the simulation.

CAN ID	*DATA* field	Target ECU
10F	02 *α*_0_*β*_0_ A0 B2 *α*_1_*β*_1_ *α*_2_*β*_2_ *α*_3_*β*_3_ *α*_4_*β*_4_	Engine
24F	44 *α*_0_*β*_0_ 1B A5 *α*_1_*β*_1_ *α*_2_*β*_2_ *α*_3_*β*_3_ *α*_4_*β*_4_	Body control
400	00 *α*_0_*β*_0_ EF 01 *α*_1_*β*_1_ *α*_2_*β*_2_ *α*_3_*β*_3_ *α*_4_*β*_4_	Display panel

### Performance Evaluation

**Fig 10:**
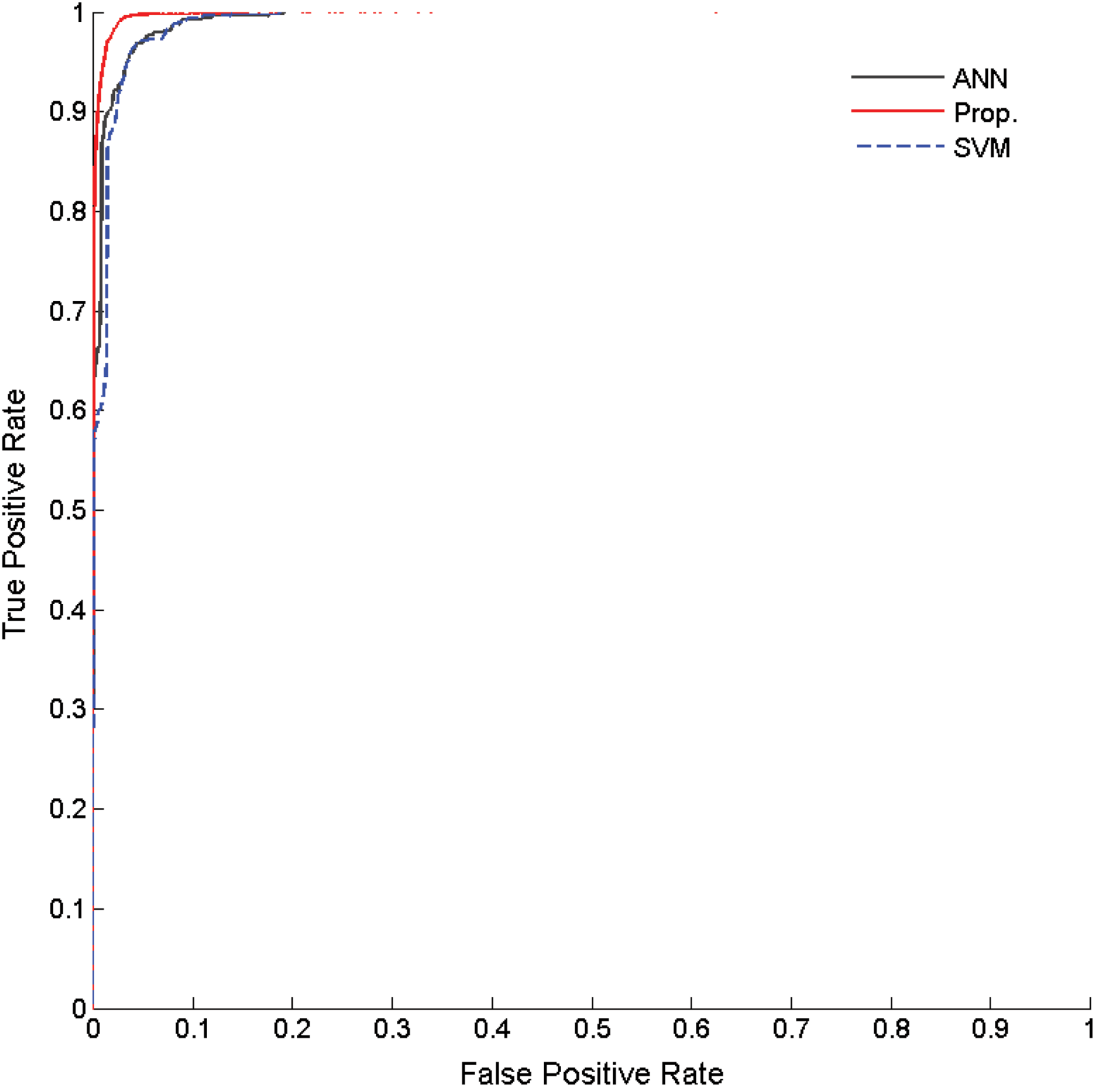
Intrusion detection performance evaluations with ROC curves.

**Fig 11:**
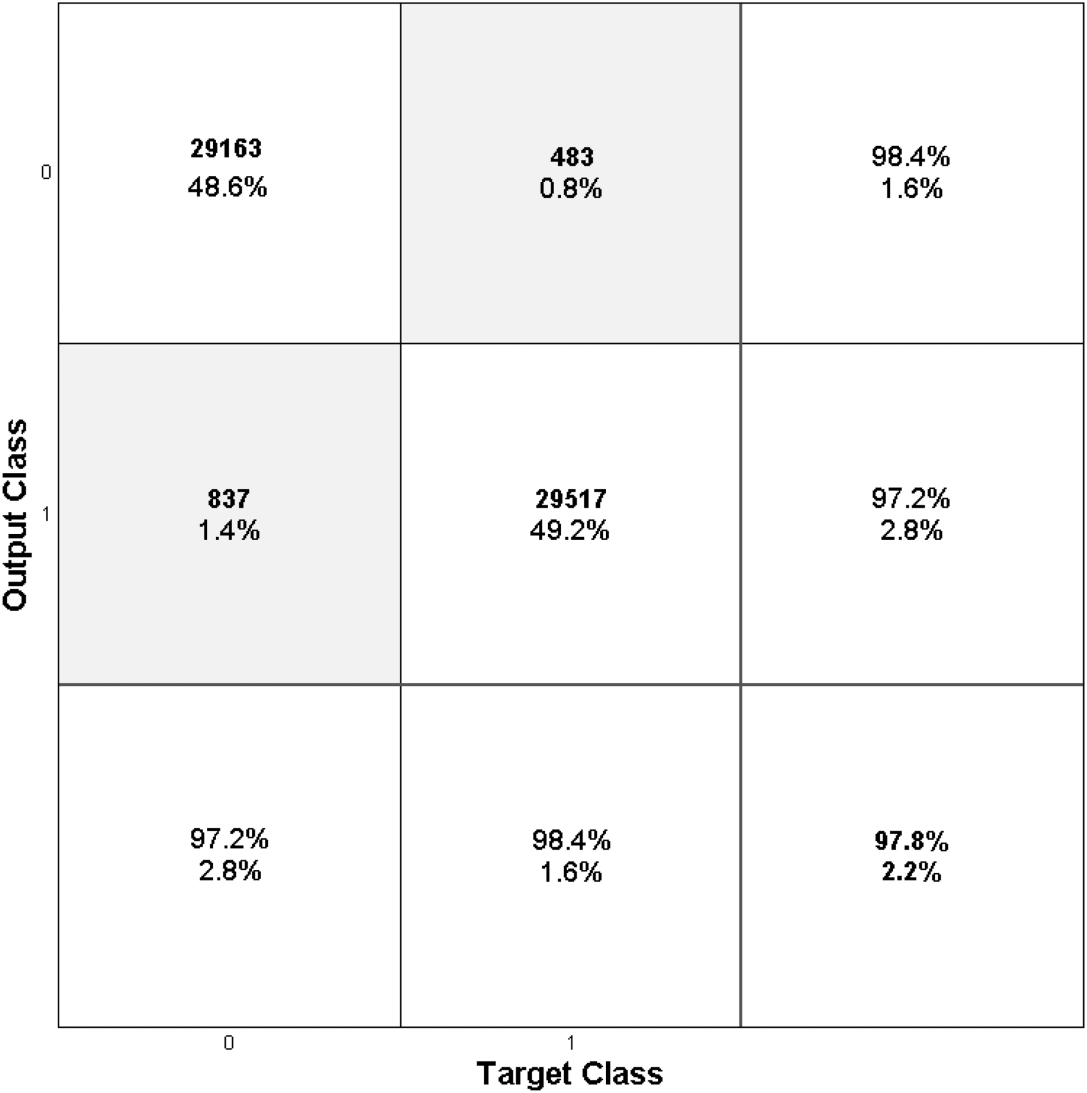
Confusion Matrix Results.

We measure the false negative rate and the false positive rate to evaluate the classification performance. *R*_*A*_ and *R*_*N*_ refer to the detection ratios of an attacking packet and a normal packet, respectively, given as,

$$\begin{array}{c} R_{A}\left(\%\right)=\frac{D_{A}}{T_{A}}\times 100, \end{array}$$ (8)

and

$$\begin{array}{c} R_{N}\left(\%\right)=\frac{D_{N}}{T_{N}}\times 100, \end{array}$$ (9)

where *T*_*A*_ and *T*_*N*_ are the total number of the attack packets and normal packets, respectively, and *D*_*A*_ and *D*_*N*_ are the number of the detected attack packets and normal packets, respectively. False positive rate should be small because it is considered more important in the attack detection. To evaluate this, we show the Receiver Operating Characteristic (ROC). The curves can be obtained by plotting pairs of the false positive rate and the hit rate with a given detection threshold, so that it provides the means to measure the trade-off between the false positive error and the correct detection. It is noted that a ROC curve shows a better detection performance when the points are ploted more in the top-left corner. Fig 10 shows the ROC curve of the proposed technique as compared to those of the artificial neural network (ANN) and the support vector machine (SVM) in the experiments. The curves clearly show that the proposed technique outperforms the conventional works in the detection ratio. The detection ratio is more than 99% when the false positive error is less than 1-2%. We also show confusion matrices in Fig 11 to evaluate the quantitative detection performances. The performance of the proposed method provides a significantly high detection ratio. The false positive error is only about 1.6%, and the false negative error is about 2.8%. The total accuracy is about 97.8%

**Fig 12:**
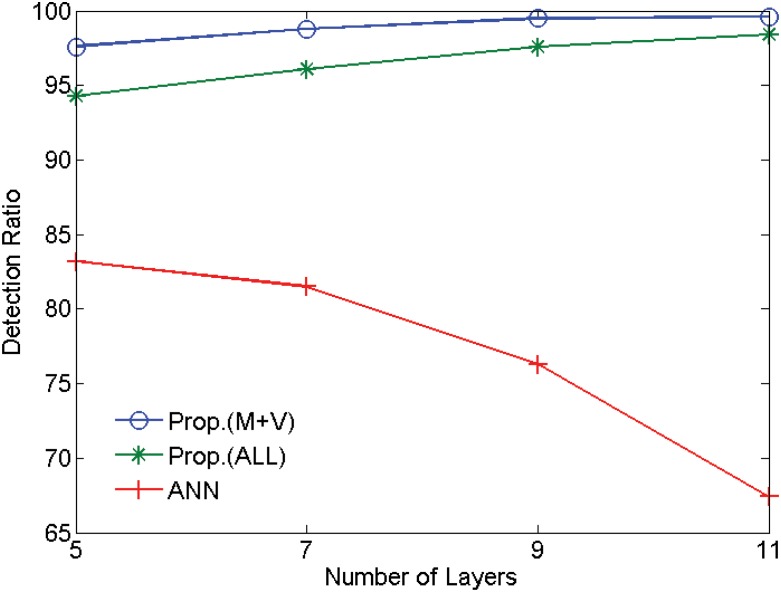
Intrusion detection performances with respect to the number of the layer.

We compare the intrusion detection performances of two variations of the proposed deep learning structure using the DNN structure to that of the conventional feed-forward artificial neural network with respect to the number of the layers. As shown in Fig 12, the proposed technique yields more accurate and consist detection performance (*R*_*A*_ + *R*_*N*_)/2 of the two scenarios than the feed-forward ANN. The ANN structure suffers from the vanishing gradient problem, causing the unstable performances with the number of the layers. For example, the lowest detection performances are observed when the number of the layer is 11. In contrast, the performance of the proposed method is significantly higher than the conventional ANN structure, but also the performance is monotonically increasing with the number of the layers. Next we show the detection performances of two variations of the proposed method. In Fig 12, DNN(ALL) presents the proposed method using a feature including all the bits (64 bits) in the *DATA* field. DNN(M+V) uses the feature including only the value information, *i.e*. *α*_*i*_ *β*_*i*_ in Table 1. As shown in Fig 12, DNN(M+V) is the best-performing method.

We also show the time complexity in the detection, depending on a different number of hidden layers in Table 2. The training time represents the measurement time needed in training the DNN structure in a training phase, and the testing time represents the measurement time in examining each packet over the network. The time complexity in a training is about 4-11 seconds, and, thus the training should be done off-line. However, the time complexity in a testing time during the packet inspection is the only 8-9 *μs* for processing features per packet and 2-5 *ms* for classifying the packets, which can be applied to a real-time application.

**Table 2:** Time complexity in a different number of layers.

Layers	Testing(*s*)	Testing	
		Feature extraction(*μs*)	Classification(*ms*)
5	4.15	8.4	2.05
7	6.32	8.5	2.29
9	9.58	8.7	3.17
11	10.81	8.7	3.78

## Conclusion

We proposed an efficient intrusion detection system (IDS) based on a deep neural network (DNN) for the security of in-vehicular network. We trained the parameters of DNN with probability-based feature vectors extracted from the in-vehicular network packets by using unsupervised pre-training method of deep belief networks, followed by the conventional stochastic gradient descent method. The DNN provides the probability of each class to discriminate normal and hacking packets, and, thus the system can identify any malicious attack to the vehicle as a result. We also proposed a novel feature vector comprising the mode information and the value information extracted from the network packets, and they are efficiently used in the training and the testing. It was demonstrated with experimental results that the proposed technique could provide a real-time response to the attack with a significantly accurate detection ratio about 98% on average when the computational complexity with the number of the layers is modestly small.

## Supporting Information

S1 File CAN packets. CAN packets generated by the OCTANE software [52].
(ZIP)
